# The dementias of schizophrenia

**DOI:** 10.1590/s1980-57642008dn10200003

**Published:** 2007

**Authors:** Ricardo de Oliveira-Souza, Rogério Paysano Marrocos, Jorge Moll

**Affiliations:** 1 Service of Internal Medicine, Gaffrée and Guinle University Hospital, Rio de Janeiro.; 2 Philippe Pinel Institute, Rio de Janeiro.; 3 LABS–D’Or Hospitals Network, Rio de Janeiro.; 4 Cognitive Neuroscience Section, National Institute of Neurological Disorders and Stroke, NIH, Bethesda, USA.

**Keywords:** dementia, schizophrenia, dementia præcox

## Abstract

Cases of “adolescent insanity” were known to Kraepelin’s forerunners and lay at the core of his concept of dementia præcox. In the post-neuroleptic era it became clear that dementia may also occur in schizophrenia as a fully reversible state depending on psychopathological status. In the present review we discuss the validity of applying the concept of dementia to schizophrenia. We concur with the view that schizophrenia may lead to a true dementia both (i) as a fixed end-stage consequence of the disease process itself, or (ii) as a drug-responsive reversible state. There is an urgent need to examine the patterns of dementia in other common neuropsychiatric disorders, employing current methods of neurobehavioral investigation.

## Abstract (pt)

Casos de “insanidade da adolescência” eram conhecidos dos precursores de Kraepelin e repousam na raiz do seu conceito de demência precoce. A era pós-neurolética deixou claro que demência pode ocorrer na esquizofrenia como estado totalmente reversível dependendo do estado psicopatológico. Na presente revisão, discutimos a validade da aplicação do conceito de demência à esquizofrenia. Concordamos que a esquizofrenia pode levar a uma demência verdadeira, tanto (i) como conseqüência do processo patológico, propriamente dito, ou (ii) como estado reversível por tratamento adequado. Fica a necessidade de se examinar os padrões de demência em outras doenças neuropsiquiátricas comuns pelos métodos atuais de investigação neurocomportamental.

## The Western mind and the concept of dementia

At certain stages of civilization, each culture develops an elaborate concept of the nature of the mind and how it relates to the worlds of spirit and flesh–*i.e.*, the organs, or bodily systems, which uphold the workings of the mind.^1^ The Western concept of the mind has its roots in Antiquity, but it was only in the XVIII century that it crystallized into a coherent theory.^2^ Accordingly, the mind is conceived as a tripartite entity composed of conation, cognition, and affectivity. Together, these compartments entail the fundamental structure of the human psyche, consisting, respectively, of the inclination to act upon the world (currently subsumed under terms such as “motivation”, “will”, and “voluntary action”), the means to attend to, store and operate upon relevant information in the environment (variably referred to as “intellect” or “cognition”), and the endowment of ideational and exteroceptive events with personal relevance, which is typically accompanied by changes in viscero-autonomic activity (described by terms such as “affect” and “emotion”). This concept has explicitly-or, more often, implicitly-framed all aspects of present-day neurological and psychiatric thought.

The concept of dementia has almost entirely fallen within the domain of cognition since its inception.^3^ Dementia was classically defined as an acquired decline of intelligence without impairment of consciousness, an idea that has been reproduced across generations of physicians with minor modifications.^4^ The American Psychiatric Association, for example, defines dementia as the association of memory impairment with at least one other cognitive deficit (such as aphasia, apraxia, agnosia, or dysexecutive) in a normally awake individual, which lead to substantial impairment in activities of daily living when compared to premorbid socio-occupational level.^5^ Obviously, memory impairment enjoys a privileged status in relation to the im­pairment of the other cognitive modalities. Moreover, the APA formulation differs in at least two aspects from previous ones. First, it does not convey prognostic implications, because it allows for the common observation that dementia is not always inexorably progressive.^6^ Secondly, nor does dementia imply a mode of onset, as one can become demented in a matter of years or minutes.^7^ This formulation does not invalidate recent attempts to define “behavioral” or “non-cognitive” symptoms of dementia^8^, provided that they continued to be considered accessory. In the present article we discuss the validity of the concept of dementia applied to schizophrenia. Written permission for the use of the pictures of the patients presented was obtained with their legal surrogates and approved by the Ethical Committee of the Gaffrée and Guinle University Hospital.

## The concept of schizophrenia: a brief overview

### Schizophrenia today: acute psychosis followed by persistent socio-occupational impairment

As currently defined, schizophrenia affects 1% of the population worldwide, representing the most devastating of all psychiatric diseases.^9^ Schizophrenia encompasses an array of clinically heterogeneous conditions with two common denominators: psychosis and socio-occupational decline.^5^ By definition, the onset of schizophrenia is marked by the emergence of psychosis. In the context of schizophrenia and allied disorders, “psychosis” refers to a syndrome characterized by hallucinations (most commonly auditory) and delusions (most often persecutory), alone or in association. After disease onset, it leads to permanent socio-occupational decline, even if psychosis remits. Once socio-occupational failure takes place, it will indefinitely remain below the individual’s premorbid level of functioning or below the level of functioning that would be expected for a healthy individual with a comparable demographic and socio-economic background. Currently, the symptoms of psychosis have been subsumed under the concept of “positive symptoms”, whereas the psychopathological manifestations that underlie socio-occupational decline have been grouped as “negative symptoms”.^10^

Several instruments with good psychometric properties have been developed for grading psychopathology and socio-occupational status of psychotic patients. The Brief Psychiatric Rating Scale (BPRS), the Scale for the Assessment of Positive and Negative Symptoms, and the Positive and Negative Syndrome Scale (reproduced in reference 10) are reliable clinical scales for the longitudinal evaluation of psychopathology, whereas the Global Assessment of Functioning Scale (GAF), which is gauged by Axis V of DSM-IV, is a unidimensional scale devised to provide an overall measure of the impact of psychopathology on everyday functioning.^5^ Investigations employing reliable instruments such as the ones just cited have confirmed that schizophrenia carries a bad prognosis. For example, complete remission was quite rare, and about 80% of a cohort hospitalized in the 1970 and early 1980’s remained disabled 6 years after their index assessment.^11^ Nevertheless, there are grounds for cautious optimism today regarding the possibility of full remission in an increasing proportion of treatment-responsive patients with schizophrenia.^12^

### The “two dementias” of schizophrenia

Depending on the particular theoretical orientation of different researchers, the psychopathological status in schizophrenia can be defined on a *categorical* or on a *dimensional basis*.^10^ The two orientations are not mutually exclusive, and each has its merits and frailties. The prevailing schemes have favored the categorical view, which was translated by DSM-IV into 5 subtypes: paranoid, catatonic, disorganized, undifferentiated, and residual. Regardless of the particular subtype, however, dementia develops more frequently in patients with “kraepelinian schizophrenia”, i.e., those who have shown continuous dependence on others for obtaining the basic necessities of daily living for more than five years despite adequate treatment.^13^ Dimensional classifications spring from factorial analyses of large numbers of patients and have provided models with 3 to 5 factors. These factors regularly include a psychotic, a disorganized, and a negative symptom cluster.

The important practical fact is that dementia in schizophrenia is often reversible with proper pharmacological treatment. The reason for this is that the kind and diversity of cognitive impairments found in schizophrenia are critically dependent on psychopathological status.^14^ As a result, large variations in cognitive impairment–ranging from dementia to none^15^–occur in any given patient depending on whether he has *active* (psychosis, thought disorder, catatonia) or *residual* disease at the time of testing.^14^ Therefore, two types of dementia, *sensu strictu*, may accompany schizophrenia as a result of the disease process: a fixed terminal dementia that insidiously ensues many years after the onset of the disease, and a potentially reversible dementia that is closely associated with psychopathology during periods of disease activity, but which tends to abate following the remission of active-phase symptoms.

For the past 12 years we have followed a cohort of outpatients with a DSM-IV diagnosis of schizophrenia in the Hospital Universitário Gaffrée and Guinle, and Instituto Philippe Pinel (IPP), two public hospitals in Rio de Janeiro. Most underwent a series of neuropsychiatric, neuropsychological and neuroimaging evaluations before and after they were enrolled in the Atypical Antipsychotics Program (AAP-IPP) supported by Dr. Ricardo Peret. The following is based on our personal experience with this demonstrative sample of patients, as well as on a selective review of the literature drawing upon only a few articles of the peer-reviewed literature to contrast them with our main arguments. Therefore, no systematic search of the literature was conducted.

### The status of dementia præcox in the era of schizophrenia

Dementia is no longer an obligatory symptom of schizo­phrenia. However, this was not always so. Indeed, the first successful taxonomic scheme envisaged dementia as the core defining symptom of this intriguing disease. The roots of the concept of schizophrenia–a form of dementia that set in during adolescence or early adulthood–were established by the end of the XIX century. First and foremost was the keen observation that the early dissimilar conditions such as catatonia^16^ and hebephrenia^17^ shared an unfavorable long-term prognosis. This formulation obviously depended on a thorough documentation of the natural course of apparently unrelated clinical states when viewed cross-sectionally, but which converged into a terminal dementia if enough time for observation was allowed, usually several years. This prognostic criterion led Kraepelin, from 1893 to 1904, to unify them under the rubric of “dementia præcox”.^18^

In fact, dementia in youth had already been recognized by others even before Kraepelin’s time. For example, in the early 1870’s the Scottish psychiatrist Clouston coined the term “adolescent insanity” to describe a psychotic illness that ran in families and afflicted mainly men between 18 and 24 years of age, and which proceeded into dementia in one-third of cases.^19^ Yet, Kraepelin is owed the merit of noting that dementia represented the end-stage of discrete clinical entities at their outset. One remarkable characteristic of this dementia was the preservation of orientation for time and space, which contrasted sharply with the disorientation observed in the early stages of senile dementia.^20^ Ironically, the importance of dementia as the unifying criterion of this group of diseases was not to last, for it soon became clear that many patients thus affected would never become demented. The appellation “schizophrenia” would fulfill the need for a more fitting descriptor only a few years after the inception of the dementia præcox concept.^21^ Yet, the forms and circumstances in which dementia occurs in schizophrenia remains an important subject in need of a fresh look bringing current methods of neurobehavioral investigation to bear.

### The dementia of dementia præcox (“kraepelinian schizophrenia”)

One major source of confusion concerning the meaning of dementia in schizophrenia is the alleged misuse by Kraepelin of the term dementia to refer to deterioration of socio-occupational status as a result of an impairment of volition.^22^ However, Kraepelin always used the term dementia to imply a disorder of cognition: “...at least in the great majority of cases, they lead to a more or less well-marked mental enfeeblement. It appears that this form of mental weakness (…) exhibits many features in common with other forms of dementia, such as are known to us as the result of paralysis, senility or epilepsy”(p.1).^18^

Kraepelin further stated that the failure in life of these individuals could not be explained solely by dementia: “Dementia præcox consists of a series of states, the common characteristic of which is a peculiar destruction of the internal connections of the psychic personality. The effects of this injury predominate in the volitional and emotional spheres of mental life”(p.3).^18^

The above quotes demonstrate why dementia præcox remains one of the most elegant applications of the tripartite mind concept to neuropsychiatry: it is one of the few conditions that compromise cognition, emotion, and conation independently and in different degrees. The following *vignette* illustrates some common patterns of dementia in schizophrenia.

***Case 1*** – This 21-year-old boy was brought by his mother at the end of 1999 for evaluation and treatment of suspiciousness and odd behaviors, which occurred continuously as a response to ill-structured delusions and voices of command. He had a secondary level of education and was normal until the age of 18, when he became defiant, stubborn, and aggressive. He responded poorly to several trials of typical antipsychotics. After 7 years of treatment with 700 mg/day of clozapine, his delusions and hallucinations decreased in frequency as did impact on behavior, although he has remained functionally impaired (GAF score=25/100). On exam, he established good rapport with his physicians, and complied with all requests. He was socially awkward and eager to put an end to conversation. On follow up, his BPRS total score of 49 has changed little, with a predominance of negative symptoms. He has scored 21/30 on the Mini-Mental State Exam (MMSE (23) and shown deficits in multiple cognitive domains. Thus, he completed 1/6 categories on the Wisconsin Card Sorting Test (WCST), and scored 30/54 and 0/29 on Benton’s Facial Recognition and 3D Block Construction tests, respectively (24). He scored 17/34 on the Token test (25). Blood tests and a brain CT were normal.

*Comment* – This is a typical case of “dementia præcox”, as defined by Kraepelin. According to current standards, he fulfills both a diagnosis of schizophrenia, undifferentiated subtype, and of dementia.^5^ He remains demented and functionally disabled.

To demonstrate the importance of extra-cognitive factors in the lowering of socio-occupational status, we present the case of a young man with a GAF score as low as that of Case 1, but with normal MMSE and formal memory scores. Judging by his appearance and dependence on others, one would hardly predict that this severely deteriorated individual could retain such a normal repertoire of cognitive abilities. This relative cognitive intactness contrasts with the cognitive pattern of the degenerative dementias of the elderly. As illustrated below, such patients usually give correct replies to questions on memory for recent events, and are likewise orientated for dates, places, and persons.

***Case 2*** – This 22-year-old patient was brought by his father at the end of 1998 because of suspiciousness and odd behaviors, which occurred continuously as a response to ill-structured delusions and voices of command, immediately prior to completing high-school. The psychotic symptoms responded to chlorpromazine. On exam, he was extremely shy and withdrawn, orienting his head and eyes away from the examiner. With some prompting, he eventually answered all questions, albeit laconically, and complied with all demands, including those required for a full neuropsychological evaluation. He scored 40 total points on the BPRS. Continuous stereotypies were noted on his face and trunk. His voluntary movements were odd and often exaggerated in amplitude. He moved slowly and walked with a mannerist gait. He was left-handed and fully oriented. He scored 28/30 on the MMSE, dropping two points, respectively, on the word recall and on the intersecting pentagons subtests. Despite his normal MMSE score, he was impaired in several neuropsychological domains, most notably executive performance (2 categories, 29 perseverative errors, and 3 failures to maintain set on the WCST), and scored 24/54, 5/30, and 14/29 on Benton’s Facial Recognition, Judgment of Line Orientation, and 3D Block Construction tests, respectively. He scored 21/34 on the Token test. In contrast, his verbal initiative (semantic categories in 1 minute: animals=17; fruits=13) and his ability to learn and retrieve new semantic material from memory, without delay and 45 minutes later (Enhanced Cued Recall Test, ECRT: 48/48) (26), were normal. He could barely take care of himself and scored 25/100 on the GAF. MRI showed several intracranial malformations, most prominently in the left temporal fossa, where a large arachnoid cyst had taken the place of a hypoplasic temporal lobe.

*Comment* – This is also a typical case of “dementia præcox” who met diagnostic criteria for schizophrenia, catatonic subtype (5). Somehow his multiple neuropsychological impairments were not of sufficient magnitude to lower his MMSE scores. Therefore, judging from his normal overall cognitive status (as assessed by the MMSE) and formal memory (as assessed by the ECRT) he was not demented. The most parsimonious explanation for his socio-occupational failure is an impairment in the neural systems that mediates will and emotion, which deprived him of the motivations, drives and emotional experiences that guide and sustain cognitive activity and endow it with personal relevance.

The interest in dementia as a manifestation of schizophrenia was reawakened in the late 1970’s as the newer neuroimaging techniques allowed the *in vivo* visualization of the brains of patients with neuropsychiatric diseases. An early study found substantial intellectual impairments in long-stay patients with schizophrenia in comparison to age-matched controls who also had been institutionalized for physical illnesses such as muscular dystrophy. The cross-sectional area of the lateral ventricles was larger in the schizophrenics and this increase was directly related to their cognitive impairment.^27^ The occurrence of dementia in schizophrenia and its association with ventricular enlargement and widespread cortical atrophy was confirmed and extended by subsequent studies employing standardized instruments, such as the Wechsler and Halstead-Reitan batteries.^28^ One post-mortem study of a large series of patients with schizophrenia and affective disorder that excluded those with Alzheimer’s and vascular diseases and controlled for age, sex, and year of birth, established that the ventricular enlargement seen in neuroimaging studies in schizophrenics was due to a predominant loss of tissue in the anterior and mid-temporal lobes.^29^ These changes were more marked than those found in the brains of patients with affective disorders, but less pronounced than those observed in patients with Alzheimer’s and Huntington’s disease.

Dementia is commonly found in chronic schizophrenic patients who underestimate their ages by 5 years or more, a symptom known as “age disorientation”.^30^ A profound loss of initiative and a host of abnormal involuntary movements are also usually observed in these cases. The lack of initiative falls within the larger group of “negative symptoms” outlined previously. Such patients spend hours to days in bed staring at the ceiling or sitting still in the backyard. They are surprisingly oriented, and capable of responding to simple questions and of interacting with medical personnel. Abnormal involuntary movements may be prominent in these patients as stereotypies and mannerisms distributed in the craniofacial territories (grimaces, howls, head twists), hands, and feet, or as whole body movements, such as circling a chair, kneeling and jumping before sitting.^31^

Dementia was also found in 6 out of 39 (15%) middle-aged patients with schizophrenia evaluated with the MMSE.^32^ Those who were additionally age-disoriented showed the lowest MMSE scores and the largest ventricles. A recent study found that roughly 10% (11 out of 120) of schizophrenics with ages ranging from 28 to 65 years were overtly demented and had ventricular and cortical sulcal enlargement on CT.^33^ In contrast to what is typical of progressive degenerative diseases, ventricular enlargement in schizophrenia is not progressive, being already present at illness onset (*i.e.*, when psychosis and odd behaviors first appear) or even before.^34^ Indeed, dementia in aged schizophrenics is often neuropathologically distinct from the other well known dementing diseases of old age.^35^ One distinctive hallmark of schizophrenia is that the histopathological changes, however varied and distributed, are not accompanied by gliosis.^36^ In all, these studies indicate that, in at least a sizeable subgroup of patients, schizophrenia is a static encephalopathy with widespread neuropathological changes of probable neurodevelopmental origin, which eventually evolves into dementia.

The idea that schizophrenia may lead to dementia is still debated on methodological grounds.^37^ However, from a practical standpoint, there is little doubt that at least a few schizophrenics become genuinely and irremediably demented. In such cases, dementia can neither simply be explained away as a manifestation of a primary impairment of will or emotion, nor attributed to fortuitous associations with degenerative diseases, such as Alzheimer’s disease^38^.

### Dementia in schizophrenia as a potentially reversible state

In a rather different context, dementia occurs during periods of disease activity and remits after successful pharmacological treatment. The following case is an illustration of how this may be seen in practice:

*Case 3* – A 35-year-old man was admitted to the emergency room because of uncontrolled fits of rage and abuse of cocaine, marijuana and alcohol in 1995. Since 16 years of age he had spent 16 months in 9 different psychiatric facilities due to assaultive behaviors that had developed in response to command voices. He had been treated with several neuroleptics with little benefit. When first seen by us, he scored 19/30 on the MMSE. At this stage, he had an education level of 8 years. He had a GAF score of 25/100 and his total BPRS score was 50. He was treated with clozapine in increasing daily doses up to 300 mg and improved slowly in the ensuing months. Six months later, there was no evidence of psychosis or aggressive behaviors and he was living with his relatives. He was diagnosed as suffering from schizophrenia, paranoid subtype. At this point, he scored 27/30 on the MMSE and 60/100 on the GAF. His total BPRS score had decreased to 22. He improved steadily and in 2003, graduated from Law school. He now works as a private civil lawyer in his own home, but remains lonesome and withdrawn when not working. In the past 5 years, his MMSE scores have ranged from 29/30 to 30/30, and he has achieved a stable GAF score of 80/100. He now has a diagnosis of schizophrenia, residual subtype.

*Comment* – This case illustrates how pharmacotherapy may dramatically change the natural course of such a destructive illness. More specifically, it shows the conversion of active into residual disease by clozapine. Following recovery from psychosis, his dementia also fully reverted, as shown by the normalization of the MMSE scores.

The dependence of dementia on psychopathology was first described by Kiloh, who called it “pseudodementia” in patients with melancholic depression.^39^ The tag “pseudo” was added to imply reversibility of dementia following normalization of mood (at the time, irreversibility and inexorable worsening were considered to be necessary characteristics of dementia). Later, it was argued that, at least while it lasted, pseudodementia was, after all, a real dementia. Therefore, the expression “dementia of depression” has been increasingly used to refer to this cognitive syndrome.^40^ Today we recognize that different patterns of dementia accompany common diseases and, accordingly, we speak of the “dementia of schizophrenia”, the “dementia of Alzheimer’s disease”, the “dementia of hypothyroidism”, and so forth.^41^

### Cognitive impairment in schizophrenics without dementia

In an attempt to establish to what extent schizophrenics are neuropsychologically normal, an oft-cited study reported the results of the assessment of 171 patients using a comprehensive neuropsychological battery. The authors found that 47 patients (27%) were neuropsychologically normal.^15^ These patients had comparatively less negative symptoms, socialized more frequently, and had not been recently admitted to a psychiatric facility. Subsequent inves­tigations established that, even when schizophrenics score in the normal range of neuropsychological performance, their scores are still lower than those of healthy controls matched for Full-Scale IQ, age, and education.^42^ These studies established beyond doubt, that cognitive impairment is intrinsic to schizophrenia. The main neuropsychological pattern is characterized by impairments in attention, executive performance, learning and memory with relative sparing of verbal knowledge and complex visual perception.^32^ Major challenges for future studies include (i) a better understanding of the patterns of dementia and cognitive impairments in schizophrenia,^43^ and (ii) how they relate to psychopathological status, (iii) how they adversely affect socio-occupational functioning, (iv) how they may differentially respond to pharmacological and cognitive-behavioral treatment,^44^ and (v) how schizophrenia can improve our understanding about the cerebral organization presiding the human mind and behavior.^45^
